# Routing Algorithms for SDM Flexible Optical Networks

**DOI:** 10.3390/e26110928

**Published:** 2024-10-30

**Authors:** Ireneusz Olszewski, Ireneusz Szcześniak, Bożena Woźna-Szcześniak

**Affiliations:** 1 Faculty of Telecommunications, Computer Science and Electrical Engineering, Bydgoszcz University of Science and Technology, S. Kaliskiego 7, 85-796 Bydgoszcz, Poland; 2 Institute of Computer and Information Sciences, Czestochowa University of Technology, J. H. Dabrowskiego 73, 42-200 Czestochowa, Poland; ireneusz.szczesniak@pcz.pl; 3 Department of Mathematics and Computer Science, Jan Dlugosz University, Armii Krajowej 13/15, 42-200 Czestochowa, Poland; b.wozna@ujd.edu.pl

**Keywords:** elastic optical networks, crosstalk per slot, routing, modulation, core allocation

## Abstract

This paper considers the online routing, modulation, spectrum, and core allocation problem in elastic optical networks. Three algorithms are proposed to minimize the bandwidth blocking probability while taking into account the impact of interference from established lightpaths. These algorithms are based on a sequence of alternative paths, ordered in terms of increasing lengths, determined by Yen’s algorithm. Each of the algorithms for the selected single or dual lightpath enforces the constraints of spectrum continuity, contiguity of the slots, and non-overlapping of the spectra with the established lightpaths, maximizes the number of bits per symbol, and selects a specific core on each link of the path. Simulation studies performed for two sample networks showed that the crosstalk has a significant impact on the bandwidth blocking probability. The best results were obtained by iterating over all paths and all slots, selecting the lightpath with the lowest average crosstalk per slot, consisting of the most loaded cores.

## 1. Introduction

The dynamic increase in traffic, caused by the exponential growth of Internet traffic and services such as high-definition television, video-on-demand, and cloud computing services [1], requires reliable transport networks capable of supporting such large traffic while maintaining network scalability. Current networks, based on Wavelength-Division Multiplexing (WDM) technology, do not fully utilize their own resources due to a coarse frequency grid that imposes a coarse-grained nature on bandwidth allocation. The mismatch between the requests of different applications and a rigid frequency grid leads to the loss of some spectrum in the network. To make more use of network resources, Elastic Optical Networks (EONs) based on Orthogonal Frequency Division Multiplexing (OFDM) have been proposed. This type of multiplexing offers the flexibility to transmit signals at widely varying binary bit rates, in the order of Gbps, over specific units of spectrum called slots. This leads to minimization of lost network resources, resulting in better network utilization compared to WDM networks. An incoming request to the EON requires the set up of a lightpath, which consists of the required number of adjacent slots with the same indices on each path link between the considered pair of nodes. The continuous increase in traffic offered to the network requires periodic increases of network capacity. Therefore, Space-Division Multiplexing, (SDM), based on multicore fibers, has been proposed in flexible optical networks [2,3]. The increase in the number of cores in fiberoptic cables, on the one hand, provides an increase in network capacity, while on the other hand, causes routing and spectrum allocation problems.

As in single-core networks, a spectrum continuity constraint must be ensured, which is to maintain the same frequency slots in all path links. However, this is where the problem arises, related to the selection of the right core in each path link. It is assumed that the cores in the individual path links can not be switched during the lightpath. In turn, the slots’ contiguity constraint requires the slots to be contiguous along the entire length of the lightpath. In contrast, the non-overlap spectra constraint precludes the use of the same spectrum (slots) by different lightpaths.

Another aspect that should also be considered when allocating demand in an SDM network is the modulation format used, which uniquely determines the number of bits per symbol. A higher number of bits per symbol provides a higher bit rate of the signal per slot (higher spectral efficiency); however, this is the price of a smaller signal transmission reach. In addition, as the number of bits in the modulation format used increases, the probability of error in its decoding increases [4]. Thus, the routing and spectrum allocation problem under consideration in SDM networks should be referred to as the Routing, Modulation, Spectrum, and Core Allocation (RMSCA) problem [5,6,7,8].

In dynamic network operation, unlike static, requests arrive randomly and the set of requests is not known in advance. Therefore, solving the RMSCA problem for each request ensures better network utilization by minimizing the bandwidth blocking probability.

In dynamic network operation, incoming requests occupy bandwidth on the network for the duration of the lightpath and then it is released as it disconnects, creating unused pieces of bandwidth at a given time. This phenomenon, known as spectrum fragmentation, results in lower utilization of network resources. To counteract this phenomenon, in the absence of spectrum to set up a request on a single path, it has been proposed to break up all traffic stream into multiple paths [9,10]. This approach improves spectrum utilization in the network; however, it introduces a differential delay in information arrival time between the first and last paths [11]. Solving this problem requires choosing such paths that the differential delay is within acceptable limits [11].

The introduction of multicore cables, on the one hand, significantly increases the capacity of the network, but on the other hand, it can cause crosstalk between link cores. The crosstalk results from the interference of signals in neighboring cores and its level depends on the distance between them. The allocated signal on specific core slots may be interfered with already occupied slots with the same indices on neighboring cores [12]. Therefore, when allocating a request, the level of possible crosstalk from slots on neighboring cores should be taken into account. If the level of this crosstalk is too high certain slots should be considered as unavailable, which may result in increased spectrum fragmentation in the network.

This paper proposes three routing algorithms, based on Yen’s algorithm [13], to solve the dynamic RMSCA problem. The first algorithm solves the RMSCA problem by taking into account two different admissible crosstalk levels affecting the allocated signal. The second algorithm solves the problem by minimizing the average level of crosstalk affecting the allocated signal on the lightpath, while maintaining an acceptable level of crosstalk on each link of the lightpath. The third algorithm, like the second, solves the problem by minimizing the average crosstalk while maintaining acceptable crosstalk level on each link of the lightpath by selecting available slots from the most loaded core on successive links of the path. Unlike other algorithms [6], these algorithms determine two paths in the same iterative loops to minimize the amount of computational effort. The proposed algorithms, using adaptive modulation, ensure that the transmission rate per single slot is maximized and the required number of slots is minimized. Extensive simulation studies have been conducted to demonstrate the effectiveness of the studied algorithms.

Since the effect of the size of crosstalk from the established lightpaths in the network on the amount of blocking probability is not sufficiently studied in the literature [5,6,7], and hence, it was the motivation for proposing these algorithms. Therefore, the proposed algorithms are the original contribution of this article.

The remainder of this paper is as follows. Section 2 of the paper presents an overview of algorithms that solve the RMSCA problem. Section 3 defines the optimization problem under consideration and the assumptions made. Section 4 discusses the heuristic algorithms proposed by the authors. Section 5 discusses the simulation results obtained, while the sixth section presents the final conclusions.

## 2. Related Works

The paper [5] proposed two new strategies to solve the RMSCA problem. The first of them is: First Core Best Fit, while the second is: Least Loaded Core Best Fit. In both strategies, a set of candidate paths is selected for each request. The first algorithm, after finding the first required set of slots, iterates further through the paths, slots, and cores to find the first best fit, leading to a reduction in core fragmentation. The second algorithm leads to finding the best fit of the required set of slots on the least loaded core. However, neither of these algorithms takes into account multi-path routing in the absence of a single path and does not take into account crosstalk between adjacent link cores.

Ref. [6] proposed the PANORAMIC algorithm, which, for an incoming request, tries to find one path and, if this is impossible, tries to find dual path. During the selection of dual path, the algorithm takes into account the differential delay, resulting from the difference in delays on the two paths. The proposed algorithm uses a spectrum mapping scheme that locates free and adjacent slots that can handle the request. Admittedly, in the article, the authors determine the amount of crosstalk per slot depending on the load for each network under study, but the proposed algorithm does not consider the crosstalk during lightpath selection.

In [7], four algorithms were proposed to solve the RMSCA problem, which were based on two image processing techniques: Connected Component Labeling (CCL) and Inscribed Rectangles Algorithm. These techniques allow efficient identification of available slots and ensure low computational complexity of the proposed algorithms. The Image-RCMLSA algorithm uses the CCL technique along with a best-fit policy. The modulation level is calculated based on the path length and estimated crosstalk. In the absence of a single path, multi-path routing is not used.

Ref. [8] proposed four new multi-path routing algorithms for the RMSCA problem based on two of the same image processing techniques, as in [7]. The techniques used are combined with different rules for matching bandwidth to a set of continuous slots in the proposed algorithms. These algorithms take into account crosstalk on allocated slots and from allocated slots to established lightpaths. Multipath routing is implemented only when there is no single path with the required bandwidth, reducing the computational effort involved in slot finding. The proposed algorithms use adaptive modulation to minimize the number of allocated slots.

Ref. [14] proposed isolated allocation of different connection types by dividing the available bandwidth resources in Flex-Grid/SDM networks. Two different strategies were compared: in the first strategy, the spectrum resources assigned to each link type correspond to the expected spectrum consumed according to the generated traffic profile, while in the second strategy, the total number of spatial channels is assigned to each link type. The paper compares these strategies, obtaining similar results for a high number of spatial channels and similar percentages for different types of connections.

## 3. Considered Optimization Problem and the Assumptions Made

The paper assumes that the network is given by a graph G(N,E), where *N* denotes a set of nodes, and *E* denotes a set of fibers (links), each of which consists of *T* cores. Let *D* be the set of real link lengths $d_{ij}$, i,j∈N, and $F=f_{1},f_{2},\ldots ,f_{\left|F\right|}$ be the set of slots supported by the transmission system on each link core. Let *g* denote the guard band between two adjacent OFDM signals.

Network nodes are spatially reconfigurable add/drop multiplexers equipped with wavelength-selective switches with transceivers with multiple inputs and multiple outputs. Each incoming request with a capacity of *C* b/s from node *s* to node *d* is denoted by a triple (s,d,C).

The solution to the RMSCA problem, minimizing the probability of bandwidth blocking, for each incoming request includes a single lightpath between a pair of *s*, *d* nodes, or if that is not possible a dual lightpath. Each lightpath is established on the basis of a sequence of cores, each of which belongs to a specific path link. Crosstalk from neighboring cores must be taken into account on each link during single or dual lightpath selection. The required number of slots for a single or dual lightpath is determined by the equation:

(1) n=⌈C/12.5m⌉+g

where *m* is the modulation level for a given modulation format, while *g* is the guard band between adjacent OFDM signals on the link.

The denominator of this expression is the bit stream, in Gbps, carried by a single slot for a modulation format with *m* bits per symbol.

The obtained solution must satisfy the spectrum continuity constraint, the slots’ contiguity constraint, and the non-overlap spectra constraint.

Whether the solution is a single lightpath or dual lightpath to minimize the amount of bandwidth occupied in the network, the modulation level on each path must be maximized. A higher modulation level requires fewer slots for the same bit stream equal to *C* b/s. The RMSCA problem under consideration is an NP-hard problem, and hence, it requires special methods. The suboptimal solution should ensure that for each request, the probability of bandwidth blocking is minimized depending on the network load.

## 4. Heuristic Algorithms

The paper proposes three new iterative algorithms that iterate over paths, slots, and cores. In the first of these, referred to as First Core First Slot (FCFS_x), the first lightpath is selected for which the average Crosstalk per Slot (CpS) on each core coming from established lightpaths on adjacent cores is not greater than the threshold. The second algorithm, named as Least Crosstalk per Slot (LCpS_x), selects the lightpath with the least CpS coming from established lightpaths, assuming that the CpS on each core of the path is not greater than the threshold x. In the last, third algorithm, named Maximum Loaded Core Least Crosstalk per Slot (MLCLCpS_x), a lightpath is selected similarly to LCpS_x; however, only one available and most loaded core is selected on each link of the path.

**Figure 1:**
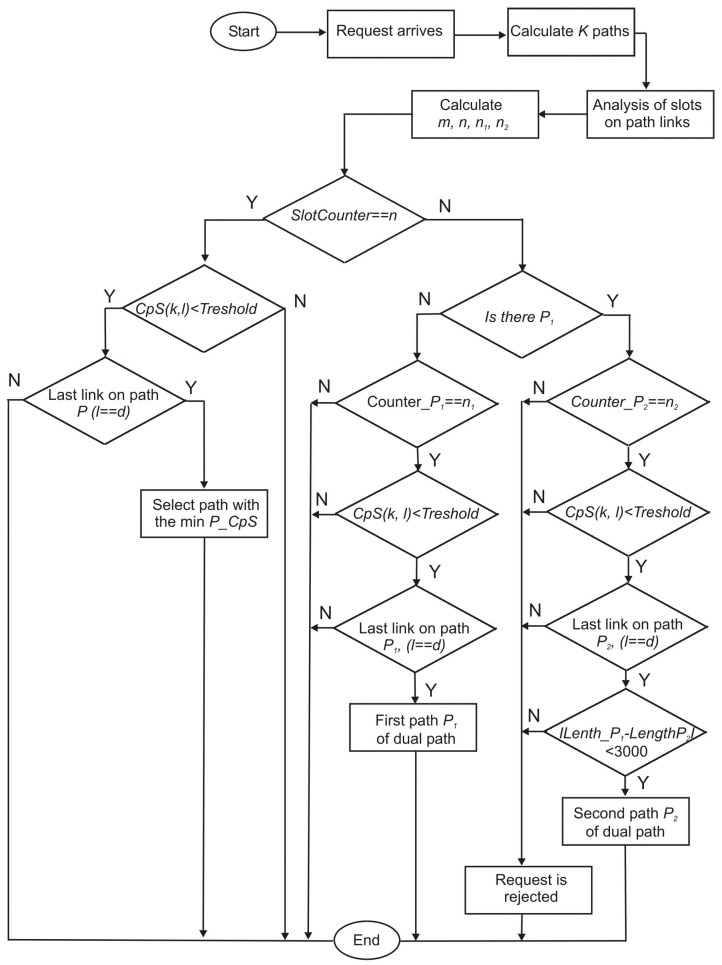
Flowchart of the proposed algorithms.

Figure 1 shows a flowchart of the proposed algorithms, which are based on Yen’s algorithm [13]. For each incoming request (s,d,C) Yen’s algorithm determines a sequence of alternative paths $Path_{i}$, i=1,…,K, ranked non-decreasing in length. Based on the number of bits *m* and Equation (1), it is possible to determine the required number of slots *n* for a given request (s,d,C), on the designated path. For a pair of paths, the total stream *C* b/s is divided into two equal parts [15]. For each of them, the required number of slots $n_{1}$ and $n_{2}$ can be determined, respectively. On the specific cores of the $Path_{i}$ under consideration, the analysis of the availability of slots is carried out. For each core, after checking the availability of the required number of *n* slots, the average Crosstalk per Slot (CpS(k,l)) on the link (k,l) is determined. This metric is defined as the quotient of the number of occupied slots ∑α on adjacent cores to the considered core to the number of occupied slots ∑β, on all cores in the considered range of *n* slots. This metric is analogous to the metric (2) in [6]; however, in this case, the averaging follows *n* slots. If the determined value of CpS is equal to or less than the Threshold for a given algorithm, then the specified slots are assumed to be available on the considered core.

Once the cores on the subsequent links of the path are established and the destination node is reached (l==d), the selection of the path is terminated. In the case of FCFS, this is equivalent to the termination of the algorithm, while in the case of LCpS and MLCLCpS further iteration is realized over successive slots and next $Path_{i}$ to find the lightpath with the lowest Crosstalk per Slotaverage P_CpS for a single path.

Unlike other algorithms, e.g., in [6], the proposed algorithms, in addition to the single path *P* selected for the traffic flow equal to *C* b/s, determine simultaneously dual path in the same iteration loops, where each of paths is determined for half the traffic, i.e., (C/2) [15]. The determined paths need not be either node-disjoint or link-disjoint. After the first path of dual path is determined for (C/2) b/s traffic, a second path is selected for the same traffic if the differential delay between the two paths is less than 15 ms, which corresponds to 3000 km in difference in the length of the two paths [11]. This approach provides a reduction in computational effort compared to the approach presented in [6]. If, after analyzing all *K* paths, the request cannot be implemented on a single lightpath *P*, then it is implemented on two lightpaths $P_{1}$ and $P_{2}$. It should be noted that the lightpaths $P_{1}$ and $P_{2}$ for a given request are selected only once in each of the proposed algorithms. Selecting consecutive lightpaths $P_{1}$ and $P_{2}$ from a set $Path_{i}$, i=1,2,…,K could result in higher bandwidth occupancy on the network. If these two paths are also missing, the request is rejected.

The pseudocodes of the proposed algorithms are shown below. The first two algorithms consist of five iterative loops, nested one inside the other, while the third algorithm consists of four loops. Iterative loops are implemented based on the while instruction. The first iteration loop iterates through the $Path_{i}$, i=1,…,K starting from the shortest path.

The second iteration loop iterates over the slots starting the iteration from slot one. The slot index determines the first slot from the set of contiguous *n* slots on each path link that are necessary to establish the lightpath.

The third iteration loop iterates over successive links (k,l) of the $Path_{i}$. Recall that each fiber (link) here contains *T* cores. Therefore, the next, fourth iteration loop, iterates over the cores of the fiber, starting with the first core.

The fifth and final loop, for a fixed $Path_{i}$, a fixed beginning slot, and a fixed core, iterates over adjacent slots, the number of which was determined after the modulation format was defined.

Assume that Slots[(k,l),core,slot] is a Boolean array informing about the availability of a slot with index slot on core core and link (k,l).

FCFS_x algorithm (Algorithm 1) selects the first lightpath *P* for which the average CpS(k,l) on each link, coming from the established lightpaths, is not greater than a threshold *x*. Therefore, iteration follows over paths $Path_{i}$, i=1,2,…,K, slots and cores up to the first available one (line 23). The term available core means that, starting from slot, there is *n* of available slots. 

**Algorithm 1** FCFS Algorithm
1:FCFS Algorithm;2:Input: G(N,E), matrix *D*, pair of node (s,d), Threshold;3:Output: *P* or $P_{1}$ and $P_{2};$4:kShortestPath(*D*, (s,d), $Path_{i},\;i=1,2,\cdots ,K$); {Determination of *K* paths by Yen’s algorithm}5:PathStatus=false; Status_P=false; $Status\_P_{1}=false$; $Status\_P_{2}=false$; $Status\_P_{1}P_{2}=false$; Best_P_CpS=∞;6:i=1;7:**while** (i≤K) **do**8: {Iterating over paths}9:  $m=m(length\left(Path_{i}\right))$;10: {calculate $n,n_{1}$ and $n_{2}$ slots on the base (1)}11: slot=1; {Initialization of the slot}12: **while** (slot+n−1≤/F/) **do**13:  {Iterating over the slots}14:  **while** $\left(k,l\right)\in Paths_{i})$ **do**15:   {Iterating over links}16:   **while** (core≤T) **do**17:    {Iterating over cores}18:    $SlotCounter=1;SlotCounter\_P_{1}=1;SlotCounter\_P_{2}=1;$19:    **while** (Slots[(k,l),core,slot+SlotCounter−1]=true) **do**20:     **if** SlotCounter==n **then**21:      **if** CpS(k,l)<Threshold **then**22:       **if** l==d **then**23:        $Status\_P_{1}=true;\;Status\_P_{2}=false;$ {Single path *P* was found. Dual Path is not necessary.}24:      **end if**25:     **end if**26:    **end if**27:    **if** $\left(SlotCounter\_P_{1}\geq n_{1}\right)$ **and not** $\left(Status\_P_{1}\right)$ **then**28:      {Choosing the first path $P_{1}$}29:      **if** CpS(k,l)<Threshold **then**30:       **if** l==d **then**31:        $Status\_P_{1}=true;$32:        $Status\_P_{2}=true;$33:       **end if**34:      **end if**35:     **end if**36:     **if** $\left(SlotCounter\_P_{2}\geq n_{2}\right)$ **and** $Status\_P_{2}$ **then**37:      {Choosing the second path $P_{2}$}38:      **if** CpS(k,l)< Threshold **then**39:        **if** l==d **then**40:         **if** $Abs(Length\_P_{1}-Length\_P_{2})<$ 3000 **then**41:          $Status\_P_{2}=false;$42:          $Status\_P_{1}P_{2}=true;$43:         **end if**44:        **end if**45:       **end if**46:      **end if**47:      SlotCounter=SlotCounter+1;48:      **if not** ($Status\_P_{1}$) **then**49:       $SlotCounter\_P_{1}=SlotCounter\_P_{1}+1;$50:      **end if**51:      **if** ($Status\_P_{2}$) **then**52:       $SlotCounter\_P_{2}=SlotCounter\_P_{2}+1;$53:      **end if**54:     **end while** {SlotCounter}55:     core=core+1;56:    **end while**; {Iteration over cores}57:    k=l58:   **end while**; {Iterating over Path links}59:   slot=slot+1;60:  **end while** {Iteration over slots}61:  i=i+1;62:**end while** {Iteration over Paths}63:**if** Status_P **then**64: **return** *P*65:**else if** $Status\_P_{1}P_{2}$**then**66: **return** $P_{1},P_{2}$;67:
**end if**
68:**if** *Status_P* **or** *Status_P*_1_*P*_2_== *false* **then**69: **return** Request rejected;70:
**end if**



The second proposed algorithm, LCpS_x (Algorithm 2), whose pseudocode is shown below, selects the lightpath *P* with the smallest average P_CpS, averaged over all path links under the assumption that the average CpS(k,l) on each link (k,l) is no greater than the threshold *x*. Therefore, iteration follows all paths $Path_{i}$, i=1,2,…,K, all slots and cores up to the first available one.

**Algorithm 2** LCpS Algorithm
1:LCpS Algorithm;2:Input: G(N,E), matrix *D*, pair of node (s,d), Threshold;3:Output: *P* or $P_{1}$ and $P_{2};$4:kShortestPath(*D*, (s,d), $Path_{i},\;i=1,2,\cdots ,K$); {Determination of *K* paths by Yen’s algorithm}5:PathStatus=false; Status_P=false; $Status\_P_{1}=false$; $Status\_P_{2}=false$; $Status\_P_{1}P_{2}=false$; Best_P_CpS=∞;6:i=1;7:**while** (i≤K) **do**8: {Iterating over paths}9: $m=m(length\left(Path_{i}\right))$;10: {calculate $n,n_{1}$ and $n_{2}$ slots on the base (1)}11: slot=1; {Initialization of the slot}12: **while** (slot+n−1≤/F/) **do**13:  {Iterating over the slots}14:  **while** $\left(k,l\right)\in Paths_{i}$ **do**15:   {Iterating over links}16:   {For MLCLCpS core= available and the most loaded core instead of a while loop in line 17};17:   **while** (core≤T) **do**18:    $SlotCounter=1;SlotCounter\_P_{1}=1;SlotCounter\_P_{2}=1;$19:    **while** (Slots[(k,l),core,slot+SlotCounter−1]=true) **do**20:     **if** SlotCounter==n **then**21:      **if** CpS(k,l)<Threshold **then**22:       **if** l==d **then**23:        **if** P_CpS<Best_P_CpS **then**24:         $Best\_P_{C}pS=P\_CpS;$25:         $Status\_P_{1}=true;\;Status\_P_{2}=false;$ {A single path was found. Dual Path is not necessary.}26:        **end if**27:       **end if**28:      **end if**29:     **end if**30:     **if** $\left(SlotCounter\_P_{1}\geq n_{1}\right)$ **and not** $\left(Status\_P_{1}\right)$ **then**31:      {Choosing the first path $P_{1}$}32:      **if** CpS(k,l)<Threshold **then**33:       **if** l==d **then**34:        $Status\_P_{1}=true;$35:        $Status\_P_{2}=true;$36:       **end if**37:      **end if**38:     **end if**39:     **if** $\left(SlotCounter\_P_{2}\geq n_{2}\right)$ **and** $Status\_P_{2}$ **then**40:      {Choosing the second path $P_{2}$}41:      **if** CpS(k,l)<Threshold **then**42:       **if** l==d **then**43:        **if** $Abs(Length\_P_{1}-Length\_P_{2})<$ 3000 **then**44:         $Status\_P_{2}=false;$45:         $Status\_P_{1}P_{2}=true;$46:        **end if**47:       **end if**48:      **end if**49:     **end if**50:     SlotCounter=SlotCounter+1;51:     **if not** ($Status\_P_{1}$) **then**52:      $SlotCounter\_P_{1}=SlotCounter\_P_{1}+1;$53:     **end if**54:     **if** ($Status\_P_{2}$) **then**55:      $SlotCounter\_P_{2}=SlotCounter\_P_{2}+1;$56:     **end if**57:    **end while** {SlotCounter}58:    core=core+1;59:   **end while** {Iteration over cores}60:   k=l61:  **end while** {Iterating over Path links}62:  slot=slot+1;63: **end while** {Iteration over slots}64: i=i+1;65:**end while** {Iteration over Paths}66:**if** Status_P **then**67: P=Best_P;68: **return** *P*;69:**else if** $Status\_P_{1}P_{2}$ **then**70: **return** *P*_1_, *P*_2_;71:
**end if**
72:**if** *Status*_*P* **or** *Status*_*P*_1_*P*_2_ == *false* **then**73: **return** Request rejected;74:
**end if**



The pseudocode of the MLCLCpS_x algorithm is very similar to the code of the LCpS algorithm, so it is omitted here. The MLCLCpS_x algorithm, unlike the previous two algorithms, does not iterate over cores, so the while loop in this algorithm is omitted (line 17 in the LCpS_x pseudocode). On each path link only one available and most loaded core is selected, where the core load is determined by the number of occupied slots. Information on the core to be selected in this algorithm is given as a comment in line 16 of the LCpS pseudocode.

This rationale about the core is derived from the Beneš algorithm for the selection of connection paths in connecting network, which, for each request, selects the connection path that passes through the most loaded and simultaneously available junctor [16].

Skipping iteration over all cores for each fixed $Path_{i}$ and each fixed slot significantly reduces the computational effort of the algorithm. This leads to reduced fragmentation of the most heavily loaded cores on the path links.

The computational complexity function still needs to be calculated. For each of the proposed algorithms, the function will be the same. First, Yen’s algorithm [13] is executed with a computational complexity function equal to O(K|N|(|E|+|N|log|N|)), where *K* is a constant and denotes the number of paths [17]. Then, in the worst case, we have five loops nested within each other for FCFS_x and LCpS_x algorithm. The first loop iterates over *K* paths, the second loop iterates over up to |F| slots in the worst case, the third loop iterates over up to |N|−1 for a path of length equal to |N|−1. The fourth loop in the worst case iterates over *T* cores, while the fifth loop iterates for the incoming request over *n* slots. Therefore, for the first two algorithms the computational complexity function of this algorithm is O(K|N|(|E|+|N|log|N|)+K|F||N|Tn). In the case of the MLCLCpS_x algorithm, we can assume that K=1. Assuming that *K*, |F|, *T*, and *n* are constant, the computational complexity function for all proposed algorithms can be defined as O(|N|(|E|+|N|log|N|)).

## 5. Obtained Results and Discussion

The chapter discusses the results obtained from the proposed algorithms. These results are compared with those obtained from the PANORAMIC algorithm [6] and the Image-RCMLSA algorithm, in which CCL-BF was used [7]. It should be noted that the PANORAMIC does not take into account the effect of crosstalk from established lightpaths on the lighpath that we search for.

**Figure 2:**
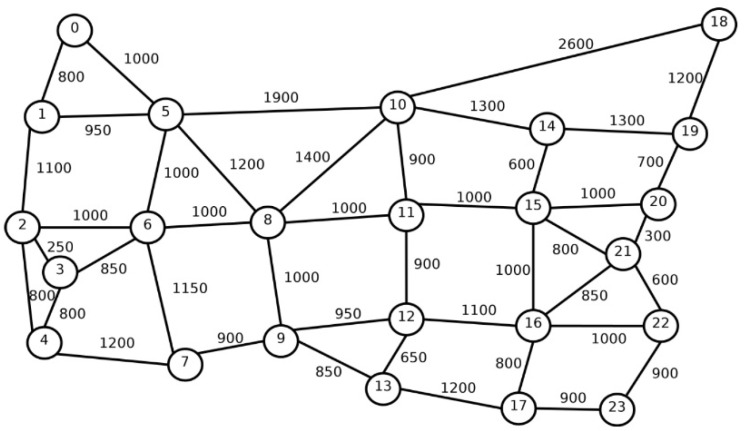
Network topology for US24.

**Figure 3:**
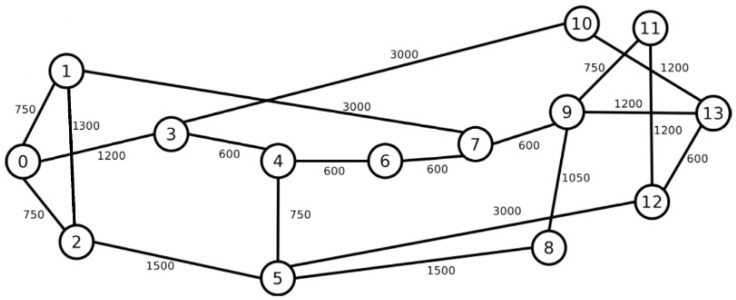
Network topology for NSF14.

Simulation studies were conducted for two different networks. The first is the US24 network, which contains 24 nodes and 43 bidirectional links, while the second is the NSF14 network, which contains 14 nodes connected by 20 bidirectional links [6]. Each link fiber contains *T*= 7 cores, arranged in a hexagonal pattern. The topological structure of US24 is shown in Figure 2, while the topological structure of NSF14 is shown in Figure 3. The graph edges of the two networks show link lengths, where each edge represents a pair of oppositely directed unidirectional links. The transmission system on each core of the network link supports 320 slots. The stream of requests offered to the network is Poisonian with an intensity of λ requests per unit time, while the duration of these requests is exponential with an average of 1/μ. Therefore, the traffic generated to the network is defined as ρ=λ/μ Erl. Table 1 shows the modulation formats used, the transmission reach for each modulation, and the traffic stream carried by a single slot [6]. The binary rate *C* for each incoming request is drawn with equal probability from the set {25, 50, 125, 200, 500, 750, 1000} Gbps. The results are recorded after the system reaches steady state, which occurs after 1000 requests have been received.

The termination of a single simulation run occurs after the arrival of 50,000 requests. The range of traffic offered to the network changes from 500 Erl to 900 Erl for the USA24 network and from 400 Erl to 900 Erl for the NSF14 network. Estimation of simulation results for each network load was made on a 30-element set. For each value obtained, 95% confidence intervals were determined.

**Figure 4:**
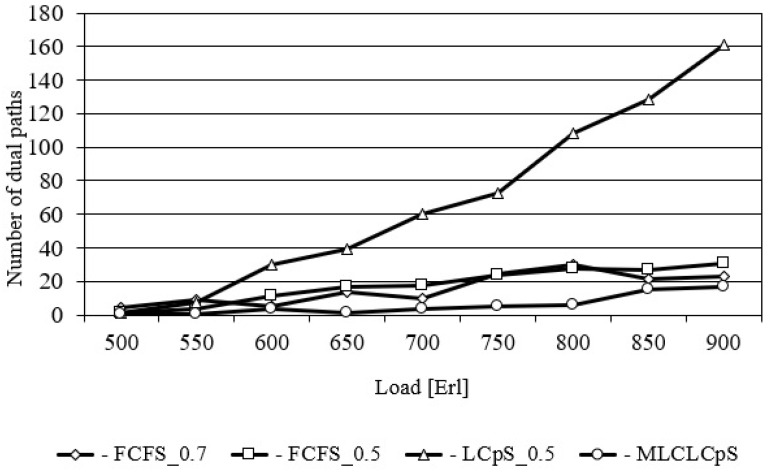
Number of dual paths for US24.

**Figure 5:**
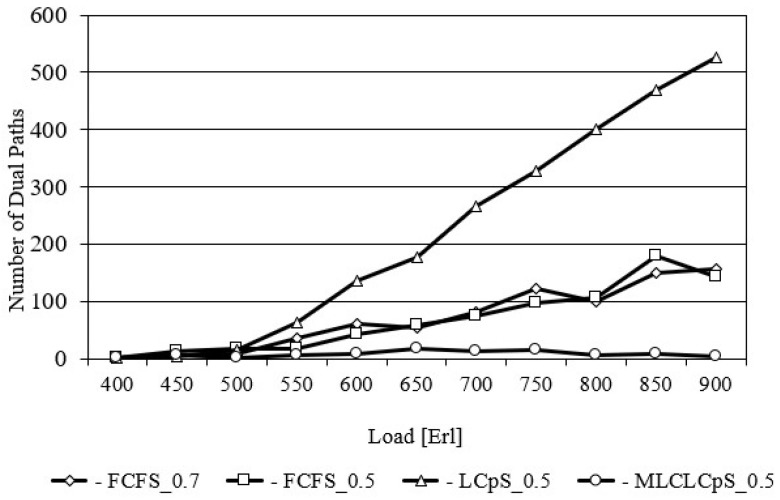
Number of dual paths for NSF14.

Figure 4 shows the number of requests established on the dual lightpaths as a function of load for the USA24 network. At this point, it should be recalled that the selection of dual path occurs only once during the selection of a single path, but the crosstalk on each core of each path must be less than the adopted threshold. This figure shows that the largest number of dual paths is used by the LCpS_0.5 algorithm for each traffic volume. This has a positive effect on minimizing spectrum degradation in the network, and thus, minimizing the probability of bandwidth blocking. In turn, the smallest number of dual paths is selected by the MLCLCpS_0.5 algorithm. For example, for a load equal to 650 Erl, the first of them selects 39.5 dual paths, while the second selects only 0.9. This is because the MLCLCpS algorithm selects the required number of *n* slots from the most loaded and available core on each pamth link. FCFS for two different values of CpS equal to 0.5 and 0.7 selects very similar numbers of dual paths for all considered network load. Figure 5 shows analogous relationships for the NSF14 network.

**Figure 6:**
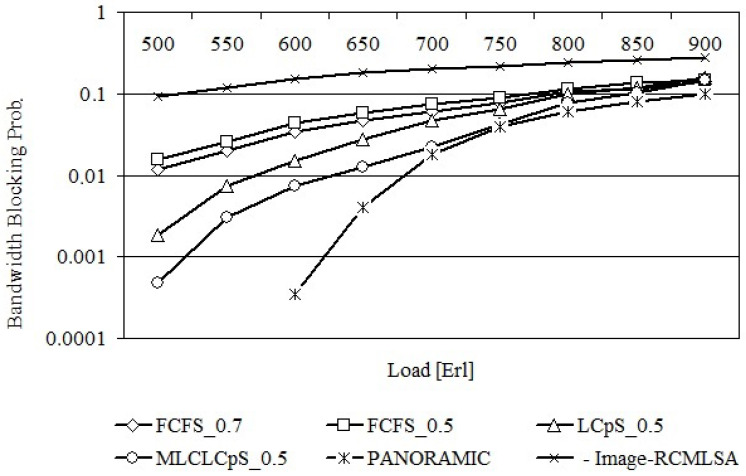
Bandwidth blocking probability for US24.

**Figure 7:**
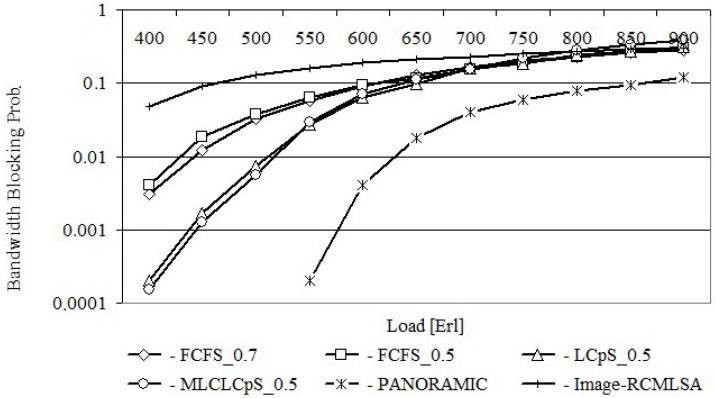
Bandwidth blocking probability for NSF14.

Figure 6 shows the bandwidth blocking probabilities for the USA24 network obtained after using each of the proposed algorithms, while Figure 7 shows the same relationships for the NSF14 network.

The bandwidth blocking probability is defined here as the ratio of bandwidth rejected to bandwidth offered to the network, i.e., $B=\sum_{i}{\bar{A}}_{i}C_{i}/\sum_{i}C_{i}$, where $A_{i}=1$ if an incoming request with bandwidth $C_{i}$ is accepted for service by the network and $A_{i}=0$ if the request is rejected.

From Figure 6, it can be seen that the crosstalk has a significant effect on the amount of bandwidth blocking probability, e.g., for a load of 650 Erl, the bandwidth blocking probability for FCFS_0.5 is equal to 0.0590 ± 0.0043, while for FCFS_0.7, it is equal to 0.0466 ± 0.0061. This difference is due to the fact that FCFS_0.7 allows a higher crosstalk of 0.7 on each core of a single or dual lightpath for the request being serviced.

Among the proposed algorithms, MLCLCpS_0.5 has the smallest bandwidth blocking probability for any network load. For example, for a load of 650 Erl, this probability for MLCLCpS_0.5 is equal to 0.0114 ± 0.0046, while for LCpS_0.5 it is equal to 0.0348 ± 0.0011. It should be noted that in the case of MLCLCpS_0.5, analogously to LCpS_0.5, the path with the smallest crosstalk is selected; however, in the case of MLCLCpS_0.5, only the single most loaded and simultaneously available core is considered on each path link, which provides the spectrum continuity constraint. This results in less spectrum degradation and reduces the bandwidth blocking probability.

Figure 7 shows the same dependencies for the NSF14 network. It should be noted that analogous dependencies were obtained as for the USA24 network; however, the differences in values between the dependencies are slightly smaller.

Figure 6 and Figure 7 still show the same relationships obtained from the PANORAMIC and Image-RCMLSA algorithms. The results obtained from the PANORAMIC algorithm represent a lower bound for the results obtained on the base proposed algorithms. However, it should be noted that the PANORAMIC algorithm does not take into account the possibility of crosstalk on the considered core coming from adjacent cores on the same link, which have a significant impact on the rejection of requests. On the other hand, the results obtained on the basis of Image-RCMLSA are significantly worse than those obtained on the basis of the proposed algorithms for any network load.

## 6. Summary and Conclusions

This paper considers the online RMSCA problem. The solution to the problem is to determine a single or dual laightpath. Each of the designated lightpaths must satisfy the constraint of spectrum continuity, the slots’ contiguity, and the constraint of no spectral overlap for adjacent lightpaths established on the cores of one or both paths. In the case of dual lightpath, the determined paths do not have to be node or edge-disjoint.

In order to minimize the occupied bandwidth in the network, it is necessary to maximize the level of modulation on each light path. The solution to the RMSCA problem also involves the selection of a core on each link of the path, which in turn can cause the phenomenon of crosstalk from adjacent link cores. The proposed algorithms should allocate the desired bandwidth to avoid crosstalk as much as possible.

Three algorithms have been proposed to solve the RMSCA problem. The first, called FCFS, iterates over paths, slots, and cores until the first single path is found. In the same iterative loops, dual lightpath is determined, which is used in the absence of a single lightpath. The amount of crosstalk on each core of a lightpath or dual lightpath must be less than an assumed threshold. Simulation studies have shown that bandwidth blocking probability is significantly lower for the larger threshold for all the considered load regardless of the network topology.

Another algorithm is LCpS, which iterates over all paths and all slots for each path to find the lightpath with the smallest crosstalk coming from links implemented on adjacent cores. In this case, the crosstalk on each link core is also below the accepted threshold.

A modification of this algorithm is MLCLCpS, which determines the single or dual lighpath by considering only the available and at the same time most loaded cores on each link of the path. This algorithm provides the lowest value of the bandwidth blocking probability.

In order to compare the solutions obtained on the basis of the proposed algorithms, the bandwidth blocking probabilities obtained on the basis of the PANORAMIC algorithm and Image-RCMLSA are shown.

Further studies should consider a larger number of paths in the absence of a single path and different traffic on each of them. However, it should be noted that as the number of paths increases, the amount of bandwidth occupied will increase.

## Figures and Tables

**Table 1:** Modulation formats used and reaches for each of them.

Modulat.	Level	Reach *l*	Bit-Rate of a
Format	*m*	[km]	Slots [Gb/s]
BPSK	1	2000 <l≤ 4000	12.5
QPSK	2	1000 <l≤ 2000	25.0
8-QAM	3	500 <l≤ 1000	37.5
16-QAM	4	250 <l≤ 500	50.0
32-QAM	5	125 <l≤ 250	62.5
64-QAM	6	l≤ 125	75.0

## Data Availability

No new data were created or analyzed in this study. Data sharing is not applicable to this article.
